# DNA and Histone Modifications Identify a Putative Controlling Element (CE) on the X Chromosome of *Sciara coprophila*

**DOI:** 10.3390/cells14161243

**Published:** 2025-08-12

**Authors:** Olga V. Posukh, Victor V. Shloma, Polina A. Skrypnik, Daniil A. Maksimov, Polina A. Antoshina, Daria A. Kalashnikova, Artem Nurislamov, Varvara A. Lukyanchikova, Nikita Torgunakov, Nariman R. Battulin, Veniamin S. Fishman, Yuri V. Vyatkin, Arina A. Smelova, Stanislav E. Romanov, Petr P. Laktionov, Daulet Valishayev, Stepan N. Belyakin, Prim B. Singh

**Affiliations:** 1 Epigenetics Laboratory, Department of Natural Sciences, Novosibirsk State University, Pirogov Str. 2, 630090 Novosibirsk, Russia; posukh@mcb.nsc.ru (O.V.P.); shloma@mcb.nsc.ru (V.V.S.); p.skrypnik@alumni.nsu.ru (P.A.S.); vift@mcb.nsc.ru (D.A.M.); polonium@mcb.nsc.ru (P.A.A.); tsun@mcb.nsc.ru (D.A.K.); smelovarina@gmail.com (A.A.S.); romanov@mcb.nsc.ru (S.E.R.); petelaktionov@gmail.com (P.P.L.); 2 Institute of Molecular and Cellular Biology SD RAS, Lavrentyev Ave., 8/2, 630090 Novosibirsk, Russia; 3 Institute of Cytology and Genetics SD RAS, Lavrentyev Ave., 10, 630090 Novosibirsk, Russia; nurislamov.ar@talantiuspeh.ru (A.N.); lukva@vt.edu (V.A.L.); n.y.torgunakov@gmail.com (N.T.); battulin@gmail.com (N.R.B.); minja-f@yandex.com (V.S.F.); 4 Scientific Center for Genetics and Life Sciences, Sirius University of Science and Technology, 354340 Sochi, Russia; 5 Department of Natural Sciences, Novosibirsk State University, Pirogova Str. 2, 630090 Novosibirsk, Russia; vyatkin@gmail.com; 6 School of Medicine, Nazarbayev University, 5/1 Kerei, Zhanibek Khandar Street, 010000 Astana, Kazakhstan; daulet.valishayev@nu.edu.kz

**Keywords:** genomic imprinting, H3K9me3, HP1, H4K20me3, heterochromatin-*like* complexes, DNA methylation

## Abstract

The term chromosomal imprinting was introduced to denote the parent-of-origin-dependent behavior of chromosomes in the fungus gnat originally named *Sciara coprophila* (current taxonomic name is *Bradysia coprophila*). Such behavior is observed in *Sciara coprophila* embryos, where paternal X chromosomes (X_p_) are specifically eliminated during the 7th–8th cleavage divisions. Elimination is regulated by a controlling element (CE) that has been mapped to heterochromomere II (H2) within the sub-telomeric short arm of polytene X chromosomes. Here, using a combination of a new *Sciara* genome assembly, along with ChIP-Seq and MeDIP analyses, we show that a 1.2 Mb region within the CE locus has a repressive epigenetic signature that is characterised by enrichments of H3K9me3, H4K20me3 and 5′-methyl cytosine (5meC). These data provide evidence for a model where the H3K9me3/HP1/H4K20me3 pathway operates to assemble a heterochromatin-*like* complex at the CE that renders it silent on X_p_ chromosomes that are not eliminated. In this regard, our findings support the idea that the H3K9me3/HP1/H4K20me3 pathway represents the most evolutionarily conserved mechanism linked to chromosomal imprinting in animals.

## 1. Introduction

Inbred stocks of the fungus gnat *Sciara coprophila* were initiated a century ago by Charles W. Metz [1]. It is a testament to his exacting studies that we have a clear picture of the extraordinary, often bizarre, behaviour of the regular and supernumerary germ-line limited or “L” chromosomes at various stages of *Sciara* development (reviewed in [2]). His work laid the foundation for research on parent-of-origin effects, now studied in many organisms from insects to man [3,4,5], where genes, chromosomes or entire chromosome sets exhibit non-Mendelian behaviour owing to their ancestry.

Non-Mendelian behaviour is observed during male meiosis in *Sciara coprophila*, where both divisions are unequal. During meiosis I the paternal set of chromosomes is eliminated. As a result, only the maternal chromosomes are incorporated into the sperm, and these same chromosomes—now of paternal origin—will be eliminated during meiosis I in male offspring. It was with regard to this reversible parent-of-origin-specific elimination of the paternal chromosome set in primary spermatocytes that Helen V. Crouse coined the term “chromosome imprinting” [6]. Meiosis II is orthodox except for non-disjunction of the paired homologs of the maternal X chromosome (X_m_) that precociously migrate to the monopole. The X_m_-dyad passes into the sperm that is now double-X (X_p_X_p_).

After fertilization, a characteristic 3X (X_p_X_p_X_m_) constitution in the zygotic nucleus is formed. Taking into account both the regular and L chromosomes, the zygote contains 11–12 chromosomes, where the contribution from the female pro-nucleus is 5 chromosomes (3 autosomes, 1 X′ or X chromosome, and 1 L chromosome) and male pro-nucleus 6–7 chromosomes (3 autosomes, 2 identical X chromosomes and 1–2 L chromosomes) [2,7].

During embryonic cleavage divisions, a striking pattern of programmed chromosome elimination occurs in the nuclei destined to form the soma. At the 5th–6th cleavage division, the L chromosomes are eliminated from all nuclei except for the germ line cells [2,8]. Furthermore, during the 7th to 8th cleavage divisions, both paternal X chromosomes (X_p_) are eliminated from somatic cells in males, while a single X_p_ is removed from the female soma. As a result, male somatic cells carry a single X chromosome (XO), whereas female somatic cells carry two X chromosomes (XX). The germ line in both sexes is XX, as one paternal X chromosome (X_p_) is eliminated from the resting germ cells on the first day of larval development. Germ-line cells retain the L chromosomes.

The elimination of the X_p_ chromosomes is regulated by a controlling element (CE) located on the X chromosome, next to the rDNA cluster in heterochromomere 2 (H2), near the X centromere. [6,9,10]. The CE not only regulates the elimination of the X_p_ chromosome in both somatic and germ cells but also controls the non-disjunction of the X_m_ chromosome presumably through centromere inactivation in secondary spermatocytes, as well as the precocious movement of the resulting X_m_-dyad toward the monopole.

How the CE controls the elimination of X_p_ chromosomes *in cis* during the 7th–8th embryonic cleavage divisions has been elucidated through detailed studies of the fate of wild-type X_p_ chromosomes and X-autosome translocations. [11]. This demonstrated that the CE does not influence the X centromere directly, but instead functions *in cis* from a distance to prevent the separation of the X_p_ arms during anaphase, causing the X_p_ chromosomes to remain at the metaphase plate and be eliminated. The elimination of the X_p_ from the germ line follows a different yet unknown mechanism that involves the passage of the X_p_ through the nuclear membrane of resting (non-dividing) germ cells [12,13].

The site and timing of the “imprints” that determine the parent-of-origin specific behaviours of the paternal chromosome set and X_p_ chromosomes have been examined through cytogenetic studies. Crouse and colleagues suggested there are two imprints, one of paternal and one of maternal origin [13]. A paternal imprint was thought to regulate the elimination of the paternal chromosome set in primary spermatocytes. In an attempt to identify the site and timing of a paternal imprint, the condensation cycles of the supernumerary L chromosomes (that are not subject to chromosome imprinting) and the regular chromosomes (that are subject to imprinting) during spermatogenesis were compared [14]. Based on these cytological observations, it was proposed that the paternal “imprint” is applied to the maternal homologues to mark them for elimination in sons during the brief interval at the end of anaphase in meiosis II, when the greatest difference is observed—L chromosomes are highly condensed (heteropyknotic) and thus likely resistant to imprinting, while the regular chromosomes remain diffuse (discussed in [14]).

A maternal imprint was indicated from the observation that the selective elimination of X_p_s during the embryonic cleavage divisions is solely under maternal control. This was shown definitively because *Sciara coprophila* is monogenic (reviewed by [2]). A given female gives rise to a family, all of which are same sex. Gynogenetic females that produce broods consisting of daughters are XX′, while androgenetic XX mothers have sons.

**Figure 1:**
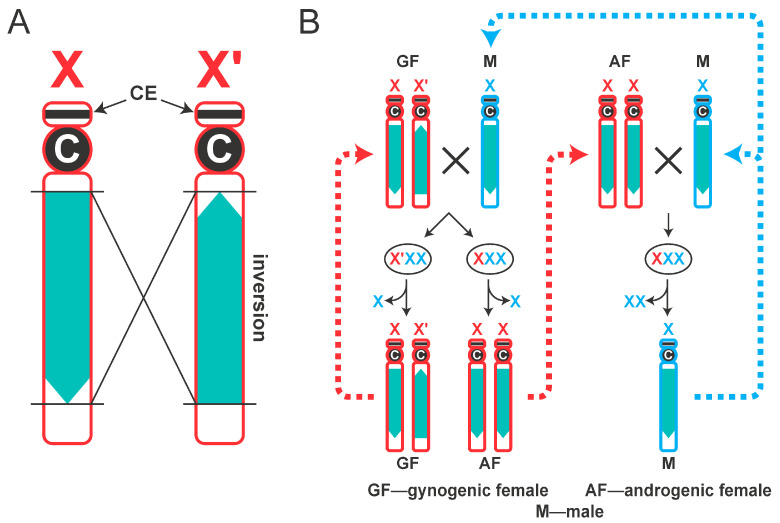
Imprinting of X chromosomes in *Sciara coprophila*. (**A**) Schematic representation of X and X′ chromosomes. A large paracentric inversion in X′ chromosome is shown in green. Letter ‘C’ designates centromere. CE position in the short arm is shown with arrows. (**B**) Behavior of X and X′ chromosomes in *S. coprophila* life cycle. X chromosomes of maternal origin (X_m_ and X′_m_) are shown in red; paternal X_p_ chromosomes are shown in blue. Gynogenic females (GF) have XX′ genotype. After mating, an aneuploid zygote containing three sex chromosomes (X_p_X_p_X_m_ or X_p_X_p_X′_m_) is formed. One of the imprinted X_p_ chromosomes is eliminated in early embryos, leading to female-only progeny–gynogenic (XX′) and androgenic (XX) females. Androgenic females lacking X′ chromosome produce eggs that are pre-programmed to eliminate both paternal X chromosomes. These females only produce males that have a single X chromosome.

The X′ chromosome possesses a long para-centric inversion (Figure 1A,B) that occurred around 0.5 million years ago [15] and is readily observable in polytene chromosome preparations as a characteristic loop between synapsed X and X′ chromosomes [16]. Accordingly, when a single wild-type male inseminates two females, one XX′ and the other XX, the outcomes are very different. This occurs because X′- and X-borne genes pre-program the cytoplasm of the fertilized egg to discard the correct number of X_p_ chromosomes during the 7th–8th cleavage in embryonic development and it is this elimination that determines whether the soma will be XX or XO (Figure 1B).

Further understanding of the nature of the maternal imprint came from studies on the X-autosome translocations used to map the CE [6,16], which proved that the egg cytoplasm does not define the sex of the embryonic soma per se, but rather determines the number of paternal X_p_s to be eliminated. Regardless of the number of chromosomes in the newly fertilized zygote, the presence of the X′ chromosome ensures that only one X_p_ chromosome is eliminated. In the eggs from XX mothers, two X_p_ chromosomes will be eliminated [6,17]. These observations suggest that, depending on the mother′s genetic makeup, any paternal imprint on X_p_ chromosomes delivered by the sperm can be recognized, disregarded, or even removed. Indeed, it was suggested that imprinting in the egg during the brief period when the parental pro-nuclei lie separately in the ooplasm is sufficient to explain all imprinting phenomena in *Sciara* [18].

**Figure 2:**
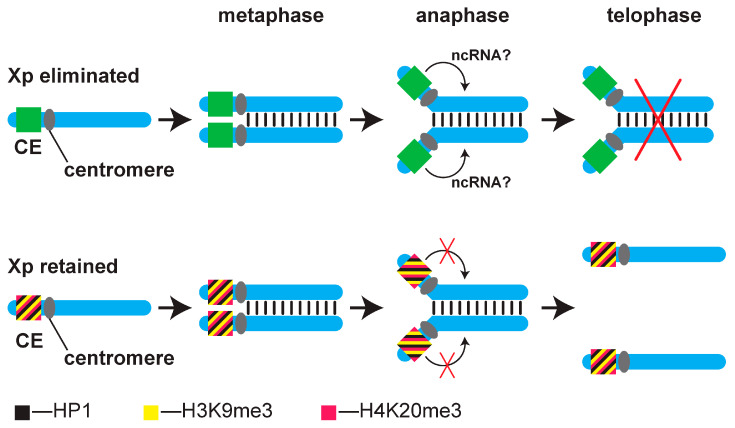
A model for the elimination of X_p_ chromosomes in the embryonic soma. Top row: the CE is expressible on the paternal X chromosome destined for elimination. Newly replicated sister X_p_ chromatids are aligned during metaphase. At anaphase, the centromeres begin to separate; however, the CE is expressed and transcribes a putative non-coding RNA that acts *in cis* to inhibit chromatid separation. As a result, the sister chromatids remain physically connected at the metaphase plate and are subsequently lost. The bottom row: in embryos that develop from eggs laid by gynogenic XX′ mothers, one X_p_ is retained. Here, the ncRNA is not expressed because the CE is embedded in the heterochromatin. The chromatids align at the metaphase plate. The centromeres and the chromosome arms then separate normally and each chromatid follows into the daughter nuclei. The model is taken and modified from [5,19].

We have proposed a mechanism by which the maternal cytoplasm regulates CE activity, thereby controlling X_p_ elimination during embryonic development. (Figure 2; [5,19]. In this model, an active CE acts at a distance to prevent the separation of the X_p_ arms during the 7th–8th embryonic cleavage, leading to their elimination. A non-coding RNA encoded by an active CE was hypothesized to undertake this function (Figure 2). In the XX′ ooplasm the H3K9me3/HP1/H4K20me3 pathway assembles a heterochromatin-*like* complex at one of the X_p_ CEs in the male pronucleus that represses CE activity [5,19]. The heterochromatin-*like* complex assembled at an X_p_ CE is epigenetically inherited through several cell divisions and preserves silencing of the CE, thereby protecting against X_p_ elimination in *cis* at the 7th–8th embryonic cleavage. Where no heterochromatin-*like* complex is assembled, the CE remains active and causes elimination of X_p_s (one X_p_ in female embryos from XX′ mothers and two X_p_s in male embryos from XX mothers).

Immuno-cytological studies on *Sciara* polytene chromosomes are consistent with the model. They showed that repressive epigenetic modifications H3K9me3 and H4K20me3, as well as the *Sciara* HP1 homologues, ScoHET1 and ScoHET2, localise to the heterochromatic region adjacent to the X chromosome centromere, where the CE locus has been mapped [5,20]. In this study, we performed de novo *Sciara* genome assembly and used it, along with ChIP-Seq and MeDIP analyses, to show that a 1.2 Mb region within the CE locus has a repressive epigenetic signature on X chromosomes that is retained, providing molecular support for the model. Remarkably, the signature also includes a unique enrichment in CpG methylation, indicating a strong evolutionary conservation of imprinting mechanisms and, further, that we have a good candidate for the long-sought-after CE locus that regulates somatic X_p_-chromosome elimination.

## 2. Materials and Methods

### 2.1. Sciara coprophila Strains and Housbandry

The HoLo2 strain of *Sciara coprophila* was used for this study (obtained from Laura Ross laboratory, University of Edinburgh). Maintenance and propagation of the colony were performed according to the procedures described on the Sciara Stock Center at Brown University web page (https://sites.brown.edu/sciara/, accessed on 22 May 2025).

### 2.2. Immunostaining of 5′ Methyl Cytosines in Polytene Chromosomes

To visualize the 5meC in polytene chromosomes of *S. coprophila*, we used the 5-Methylcytosine antibody (Active Motif, Carlsbad, CA, USA, Cat. No. 61255). Chromosome squashes were prepared from formaldehyde-fixed salivary glands of fourth instar larvae and stained with antibodies, following the procedures described in Pindyurin et al., 2008 [21]. Immunostaining was performed without DNA denaturation. To control for the specificity of the immunostaining, the negative control was performed without α-5meC antibodies (Supplementary Figure S1).

### 2.3. Sciara coprophila Genome Sequencing and Assembly

Whole genome sequencing of genomic DNA from *S. coprophila* females was performed using Oxford Nanopore (54x coverage) and Illumina (32x coverage) technologies. The data are available at NCBI BioProject PRJNA1273090. Initial assembly was achieved using the long Nanopore reads, which were subsequently improved with Illumina-derived sequences to remove the sequencing discrepancies. Final version of the genome assembly was obtained after Hi-C procedure (BioProject PRJNA1271224), which enabled the correct ordering and orientation of contigs (NCBI accession number JBPSMZ000000000). Detailed description of the procedures is presented in the Supplementary Materials and Supplementary Figures S3 and S4.

### 2.4. Methylated DNA Immunoprecipitation (MeDIP)

For methylated DNA immunoprecipitation (MeDIP), genomic DNA from *S. coprophila* was isolated from 72-h-old female embryos. The DNA was fragmented by sonication using a BioRuptor Pico (Diagenode) to generate fragments with an average length of 200–250 bp. Sequencing adapters for the Illumina platform were ligated to the fragments using the TruSeq Nano LP kit (Illumina). Ten percent of the material was reserved as input control for data normalization.

The DNA samples with ligated adapters were denatured at 95 °C for 5 min and then used for immunoprecipitation with anti-5meC antibodies (Active Motif, Cat. No. 61255), as described in [22]. The enriched fraction was treated with Proteinase K, purified by phenol–chlorophorm extraction and precipitated with ethanol in the presence of 20 µg of glycogen. The recovered DNA was amplified using primers specific to the adapters according to the Illumina TruSeq Nano protocol.

The final libraries were sequenced using an Illumina MiSeq instrument. The data are available as NCBI BioProject PRJNA1273090. The resulting reads were aligned to the assembled *S. coprophila* genome using MOSAIK software v1.1 [23] with the following parameters: -m all -mmp 0.1 -act 20 -a single. Only uniquely aligned reads were used to generate DNA methylation profiles by dividing the MeDIP data, by the input control data and normalizing by sequencing depth. Local enrichment was calculated in 1000 bp windows with a 100 bp step and is presented as the log2 ratio of MeDIP signal to input control (Log2(IP/input)). The experiment was performed in two biological replicates.

### 2.5. ChIP-Seq

Formaldehyde-fixed chromatin from approximately 20 μL of 72-h-old female embryos was used for each ChIP experiment. Fixed chromatin was fragmented using the BioRuptor sonicator (Diagenode, Denville, NJ, USA). Anti-H3K9me3 antibodies (Active Motif, Cat. No. 39161), anti-H4K20me3 antibodies (Active Motif, Cat. No. 39671) and anti-H3K4me3 antibodies (Diagenode, Cat. No. C15410003) were used with the True MicroChIP & MicroPlex Library Preparation™ Package (Diagenode), according to the manufacturer′s recommendations. Three biological replicates were processed. Enriched DNA fractions and untreated input samples were used for library preparation using the Illumina Nextera procedure and sequenced on the Illumina MiSeq instrument (paired end, 75 bp). The data are available as NCBI BioProject PRJNA1273090. The reads were aligned to the *S. coprophila* genome assembly using MOSAIK software v1.1 [23] (key parameters: -m all -mmp 0.1 -act 20 -a single), paired reads were combined. Only uniquely aligned fragments were used to generate profiles. For each genomic position, the RPM (reads per million) values were calculated in the ChIP and input samples. Log2 of the ChIP to input ratio was used as a measure of local enrichment. The resulting data were smoothed using an averaging 1-kb sliding window, step 100 bp.

### 2.6. FISH on Polytene Chromosomes

DNA fragments were amplified from *S. coprophila* genomic DNA using the following primers:

probe_L-for: 5′-GTCAGCGGATTAAAGCTCTCTAT-3′
probe_L-rev: 5′-TTGGCGAACCACTTCTACTG-3′
probe_R-for: 5′-CCATGCGTCGGTGAGTTATTA-3′
probe_R-rev: 5′-GTGACCTGAAAGTACTGAGTGG-3′

These PCR products were used for direct labeling with Tamra-5-dUTP (probe L) or FLu-12-dUTP (probe R) (Biosan, Russia) in random-primed reaction with Klenow fragment. Chromosome preparation and hybridization were performed as described in [24]. Hybridization signals were observed using the LSM 710 Confocal Microscope (Zeiss, Oberkochen, Germany).

## 3. Results

### 3.1. The Repressive Epigenetic DNA Modification 5′ Methyl Cytosine (5meC) Is Enriched at the CE

**Figure 3:**
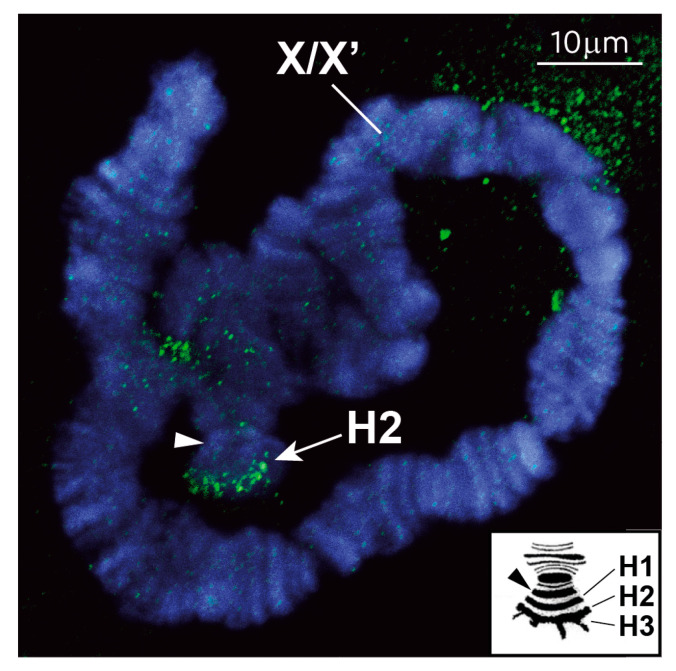
5meC DNA methylation in polytene chromosomes of *Sciara coprophila*. Paired X and X′ chromosomes from the L4 female larva form a characteristic loop due to the presence of a large inversion in the X′ chromosome. 5meC-specific antibody staining (FITC, green) reveals the presence of this epigenetic marker specifically in the region of H2 heterochromomere harboring the CE (indicated with arrow). No similar enrichment in 5meC was observed elsewhere. Chromosomes were stained with DAPI (blue). Arrowhead in photomicrograph and inset points to the centromere. Inset: schematic representation of the locations of the H1, H2 and H3 heterochromomeres in the short arm of the *S. coprophila* X chromosome (adapted from [28]).

Prompted by the observation that imprinting in many animals is crucially dependent upon the classical repressive epigenetic modification 5meC [3,4] we decided to test whether the CE is enriched in 5meC. Accordingly, we stained L4 larval polytene chromosomes with anti-5meC antibodies. We paid attention to the short arm of polytene X chromosomes that contains three sub-telomeric heterochromomeres (termed H1, H2 and H3) adjacent to the centromere. As was shown in the early studies, the CE locus is contained in the H2 heterochromomere [6]. These heterochromomeres are rarely seen in a linear arrangement (see inset in Figure 3); rather, they typically appear as an irregular mass of sub-telomeric heterochromatin [25,26,27]. As shown, anti-5meC antibodies recognize this particular sub-telomeric heterochromatin (arrow in Figure 3) adjacent to the centromere (arrowhead in Figure 3) on the X chromosome. No similar enrichment in 5meC was observed elsewhere (Supplementary Figure S2). Notably, our chromosome preparations were not subject to alkaline treatment to make the 5meC mark more accessible for antibodies, indicating that 5meC is highly enriched at the H2 CE locus.

### 3.2. New S. coprophila Genome Assembly and Analysis of the Epigenetic Marks at the CE

Immuno-cytological studies on polytene X chromosomes have shown that repressive histone (H3K9me3 and H4K20me3; [5,20]) and DNA (5meC) modifications localize to the chromosomal region where the CE maps (Figure 3). We next investigated the genomic localization of the repressive epigenetic modifications at higher resolution using ChIP and MeDIP approaches. To that end, we generated a high-resolution map of the *S. coprophila* genome because, at the time the study was undertaken, no map was available. We used Oxford Nanopore to produce a draft assembly of the genome and polished it with Illumina reads to correct for sequencing artifacts. A chromosomal-level assembly was achieved with the help of the Hi-C method that not only improved genome quality but also allowed us to confirm the presence of chromosome loops and the breakpoint positions of the X′ inversion (Supplementary Figures S3 and S4) that were recently reported by others [15]. The detailed methodology concerning *S. coprophila* genome sequencing, assembly, Hi-C, as well as the general characteristics of the assembly, are found in Supplementary Materials.

**Figure 4:**
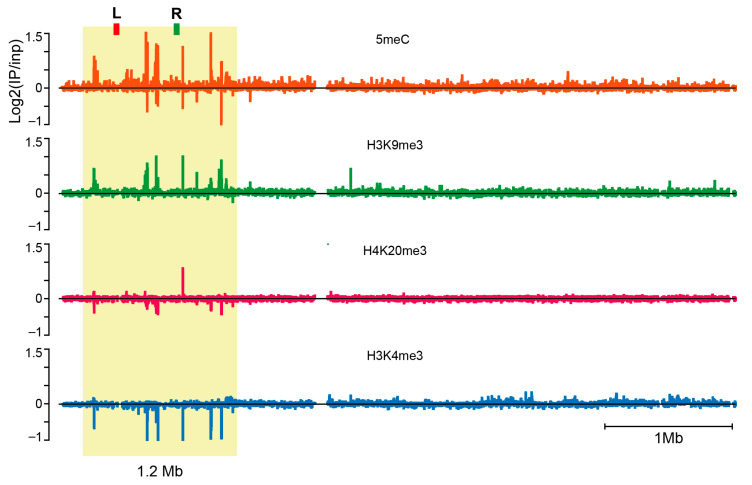
A single locus of 5meC in *Sciara coprophila* genome is associated with repressive histone modification and depleted in an “active” histone modification. A MeDIP experiment using chromatin from 72 h embryos reveals a single region in the Sciara genome that contains a series of 5meC peaks (approximately 1.2 Mb region of the PGA_scaffold_1 in our genome assembly; shaded with yellow). The locations of FISH probes (L and R) that were used to confirm the position of this region in polytene chromosomes are indicated (see Figure 5). ChIP experiments confirm the presence of heterochromatic histone modifications (H3K9me3 and H4K20me3) and depletion of the active chromatin marker H3K4me3 in the 1.2 Mb region. The data are presented as log_2_(IP/Input) values, where positive values indicate regions enriched for the corresponding epigenetic mark, and negative values indicate depleted regions.

H3K9me3, H4K20me3, H3K4me3 and 5meC enrichments were localized in the genome of 72-h gastrulating *S. coprophila* embryos. The embryos developed from gynogenic XX′ eggs and thus contained 2X chromosomes (X_m_X_p_) after one X_p_ has been eliminated at the 7th–8th cleavage division. We began with MeDIP to localize 5meC. The MeDIP profile revealed a single, unique locus in the *Sciara* genome that contains a series of 5meC peaks. As shown, the 5meC peaks were scattered within a 1.2 Mb region of a ~5Mb-long scaffold (termed PGA_scaffold_1) derived from the assembly (Figure 4, upper profile).

**Figure 5:**
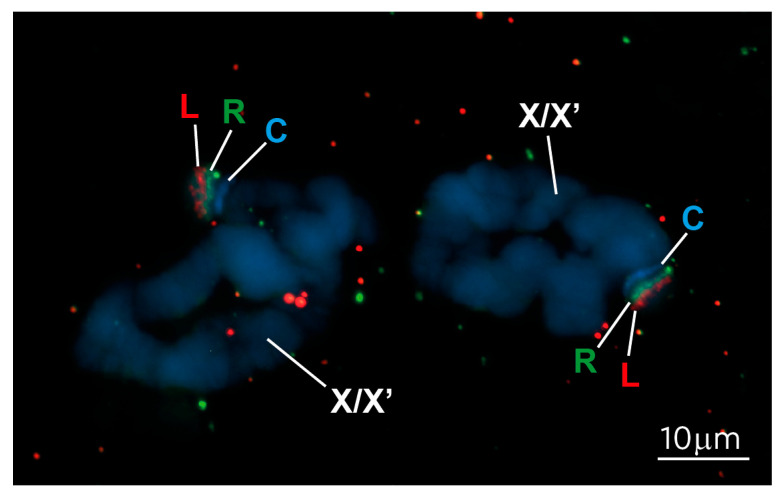
FISH mapping of the 5meC-enriched region revealed in MeDIP. The FISH probes L (TAMRA, red) and R (Fluorescein, green), schematically shown in the Figure 4, were hybridized with *S. coprophila* polytene chromosomes, confirming that the unique 5meC-enriched region from the PGA_scaffold_1 locates to the sub-telomeric heterochromatic short arm of the X chromosome, coinciding with the site of 5meC antibody staining (Figure 3). C indicates the position of the centromere in the synapsed XX′ chromosomes. Chromosomes were stained with DAPI (blue).

To confirm that the 1.2 Mb region corresponds to the sub-telomeric region of the X chromosome, where we observed the cytological enrichment of 5meC (Figure 3), we undertook in situ hybridization using non-repetitive FISH probes, called L and R (marked on the upper profile of Figure 4), from the 1.2 Mb genomic region containing the DNA methylation peaks. Hybridization of L and R to polytene chromosomes confirmed that the region containing the 5meC peaks maps to the sub-telomeric region adjacent to the centromere, where we observed the 5meC antibody staining (c.f. Figure 3 vs. Figure 5).

**Figure 6:**
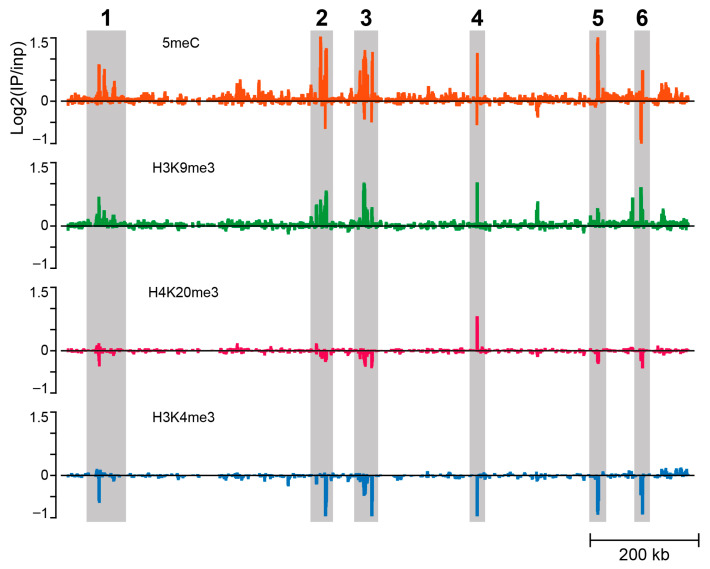
High resolution profiles of 5meC, H3K9me3, H4K20me3 and H3K4me3 in the 1.2Mb region that maps to the CE. The 1.2 Mb region (marked in yellow in Figure 4) at high resolution shows 6 distinct peaks (numbered 1 to 6) of 5meC that coincide with peaks of H3K9me3. The 6 peaks are depleted in the “active” histone modification H3K4me3. H4K20me3 shows limited alignment with repressive histone modifications, except for peak 4. The data are presented as log_2_(IP/Input) values, where positive values indicate regions enriched for the corresponding epigenetic mark, and negative values indicate depleted regions.

Profiles of H3K9me3 and H4K20me3 (profiles 2 and 3, counting from top in Figure 4) derived from ChIP-seq experiments showed that the same region enriched for 5meC also showed enrichment for these two repressive histone modifications, with H3K9me3 being more pronounced compared to H4K20me3. We also profiled H3K4me3, a histone modification associated with gene activity and antagonistic to H3K9me3 and H4K20me3 that is depleted in heterochromatic regions [29,30]. As expected, the H3K4me3 profile (bottom profile in Figure 4) shows depletion in the 1.2 Mb region. At higher resolution, six peaks of 5meC coincide with six peaks of H3K9me3; co-incidence is only observed at peak 4 for H4K20me3, where all three repressive epigenetic marks align (Figure 6). The active histone modification H3K4me3 is depleted at all six peaks (Figure 6). These data again support a role for epigenetic silencing of the CE locus in regulating the retention of the X chromosomes at the time the active CE leads to elimination of X_p_ chromosomes (Figure 2).

## 4. Discussion

Parent-of-origin-specific imprinting occurs when the parental genomes are separated. This is during gametogenesis in the male and female germ-lines and when the paternal and maternal pro-nuclei lie separately in the ooplasm of the newly fertilized zygote. The relative contributions of “germ-line” and post-fertilization “maternal” imprinting depend on the system studied [5]. Yet, as shown by this work, and a large body of evidence from classical imprinting systems in *Coccids* [18,31,32] and mammals [3,4], the mechanism(s) that confer epigenetic (i.e., cell-to-cell inheritance) differences between parental alleles are conserved.

**Figure 7:**
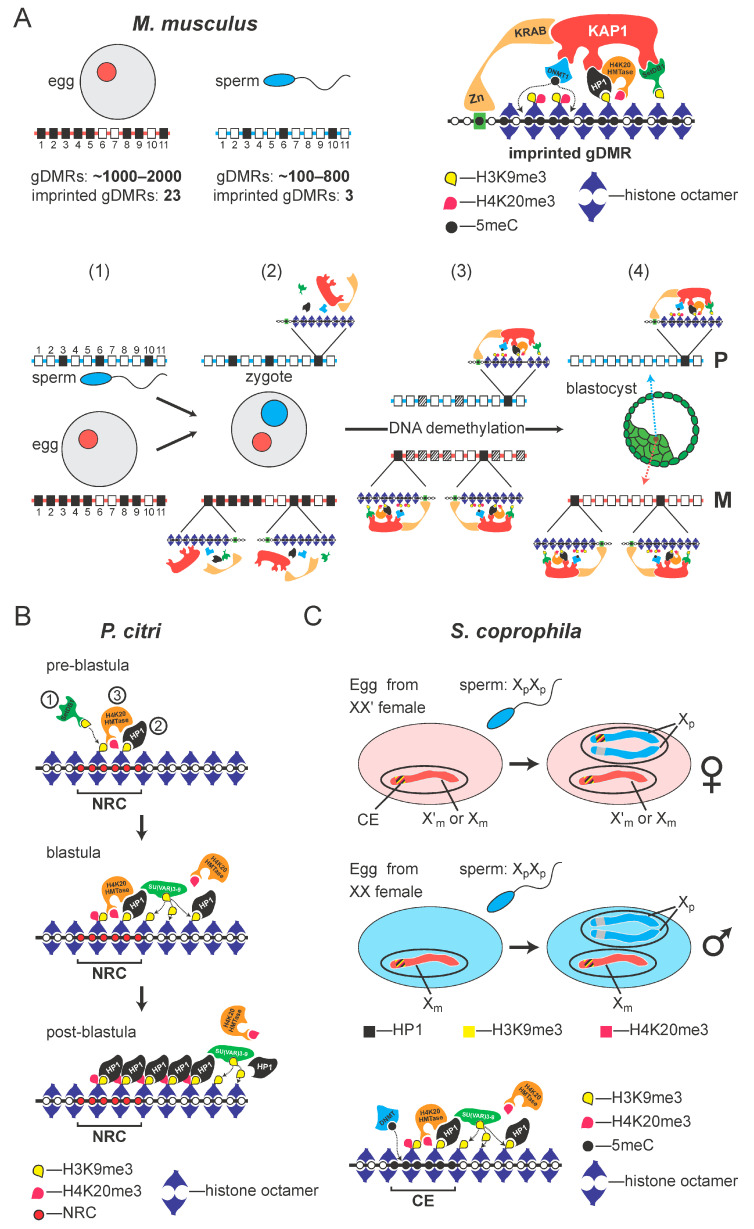
The H3K9me3:HP1:H4K20me3 pathway and the conservation of imprinting mechanisms in animals. (**A**) In the mouse, the egg nucleus (red circle) contains 1000–2000 oocyte-specific gDMRs, of which about 23 are definitive imprinted gDMRs. The sperm nucleus (blue circle) contains around 100–800 gDMRs, of which only 3 are definitive imprinted gDMRs. Below the oocyte and sperm, maternal (red line) and paternal (blue line) homologous chromosomes are presented with the CpG islands (CGIs) depicted as rectangles numbered 1 through 11. Open rectangles represent non-methylated CGIs. Some methylated CGIs (closed rectangles) are shared, e.g., position 3 on both parental homologues, and thus are not gDMRs. Many methylated CGIs are nonimprinted gDMRs (position 6 on the paternal chromosome and positions 2, 4, 5, 9 and 11 on the maternal chromosome), which will be demethylated during preimplantation development development (shown in panels (1) through to (4). Preservation of methylation at definitive imprinted gDMRs (position 10 on the paternal chromosome and 1 and 8 on the maternal homologue) is maintained by the assembly of heterochromatin-like complexes that are targeted to imprinted gDMRs by KRAB-ZNPs that recognise a hexamer motif TGCCGC found in imprinted gDMRs, where the central CpG dinucleotide is methylated (shown in panels (2) through to (4)). KRAB-ZNPs bind KAP1, which recruits the Setdb1 histone methyltransferase, HP1 and Dnmt1. HP1 binds the H3K9me3 generated by Setdb1 and recruits a H4K20me3 histone methyltransferase that generates H4K20me3, thus forming the H3K9me3:HP1:H4K20me3 pathway (given at top right in (**A**). DNA methylation at the imprinted gDMR is maintained (dotted lines) by Dnmt1. Taken from [54]. (**B**) The H3K9me3:HP1:H4K20me3 pathway also operates in coccids to maintain the silenced, heterochromatic state of the imprinted chromosome set in males. In the pre-blastula the P. citri SETDB1 H3K9 HMTase homolog (SetDB1pc1) deposits the H3K9me3 histone modification at the nuclease-resistant fraction. H3K9me3 is recognized and bound by the HP1 homolog, PCHET2. PCHET2 recruits the Hmt4-20 HMTases that generate the H4K20me3 modification at the nuclease-resistant fraction (top row). At the blastula stage, when heterochromatinization begins, H3K9me3 “spreads” along the paternal chromosome. H3K9me3 is deposited by the Su(var)3–9 pc3/4 homolog. H3K9me3 is bound by the HP1 homolog PCHET2 (middle row). In the post-blastula embryo, the H3K9me3:HP1:H4K20me3 pathway maintains the silenced, heterochromatic state of the paternal chromosomes (bottom row). Taken from [52]. (**C**) Imprinting of the controlling element (CE) in Sciara involves the H3K9me3:HP1:H4K20me3 pathway that assembles a repressive heterochromatin-like complex at the CE, rendering it inert. As shown, the haploid maternal nucleus laid by the XX′ mother contains either an X or X′ chromosome. The maternal CE on the X or X′ chromosomes is rendered inert by the H3K9me3:HP1:H4K20me3 pathway that assembles a heterochromatin-like complex. After the fertilization by the double-X (X_p_X_p_) sperm, the paternal pro-nucleus is formed and conditioning of the ooplasm by the XX′ mother results in assembly of a heterochromatin-like complex at one of the two paternal CEs, rendering it inexpressible, like the maternal CE; the complex is assembled at the CE on the X_p_ that is retained. The remaining paternal CE is an “open” expressible state and X_p_ is eliminated. The bottom row depicts an egg laid by the XX mother. The maternal CE is again inexpressible due to the assembly of a heterochromatin-like complex, while both paternal CEs are in an “open” expressible state; both X_p_s will be eliminated. The stripes of colour at the CE represent H3K9me3 (yellow), H4K20me3 (purple) and HP1 protein (black). Female chromosomes are in red and males’ are in blue. Modified from [5]. At the bottom of (**C**) is the H3K9me3:HP1:H4K20me3 pathway that operates at the imprinted CE. H3K9me3 found that the CE (Figure 4 and Figure 6) is generated by a K9HTMase and is bound by Sciara HP1 homologues that are known to localise to the CE [20]. H4K20me3 present at the CE (Figure 4 and Figure 6) is generated by an H4K20MTase that is recruited through an interaction with HP1. CpG methylation at the CE is generated by a DNA methyltransferase (DNMT), which indicates that imprinting in Sciara is similar to that observed in mammals (panel (**A**)).

In the mouse, DNA methylation (5meC) in the respective germ-lines plays a crucial role in “marking” the parental genomes, but specificity, the need to specifically preserve the methylation “mark” at “imprinted” genes is under maternal control [33]. In mice, there are around 1000–2000 oocyte-specific germline differentially-methylated regions (gDMRs) and 185–818 sperm-specific gDMRs [34,35]. Of all these gDMRs present in the pro-nuclei [35], only 26 imprinted gDMRs retain methylation after the global DNA demethylation that occurs during the pre-implantation stage of embryogenesis [33,36]. The methylation of DNA at these imprinted gDMRs is preserved by a mechanism involving the sequence-specific KRAB zinc-finger proteins (KRAB-ZFPs), ZFP57 [37,38] and ZFP445 [39]. For example, ZFP57 binds the TGCCGC motif found in imprinted gDMRs, only if the central CpG dinucleotide is methylated [38,40,41]. The binding of KRAB-ZNFs results in the local assembly of a heterochromatin-*like* complex (Figure 7A) that includes the universal co-repressor of KRAB-ZNFs, KAP1, which directly interacts with Dnmt1 [38,42], thereby ensuring maintenance of 5meC. KAP1 also recruits the SETDB1 K9 histone methyltransferase (HMTase) that generates H3K9me3 that is bound by HP1, which, in turn, can recruit H3K20 HMTases to generate H4K20me3 [43,44]. This H3K9me3/HP1/H4K20me3 pathway is conserved and plays a role in imprinting in coccids [45,46,47,48] (Figure 7B).

In coccids there is evidence that imprinting takes place post-fertilization and is under maternal control [18,31] (Figure 7B). Studies on coccids and closely related soft scale insects that produce males and females without contribution from the father [49,50,51] led to the conclusion that the ooplasm regulates heterochromatinization after fertilization, by imprinting the paternal chromosome set prior to syngamy, while it is separated in the pro-nucleus [18,31]. This imprint on the paternal set is epigenetically inherited through the rapid cleavage divisions when both chromosome sets are euchromatic. Later, at the blastula stage, this imprint causes selective heterochromatinization of the paternal chromosome set in male embryos. Our work on coccids is consistent with this scenario [52]. The imprint is maintained by the H3K9me3/HP1/H4K20me3 pathway that forms localised heterochromatin-*like* complexes on the paternal chromosomes during the early cleavage divisions and nucleates spreading of heterochromatinization on the entire paternal chromosome set at the blastula stage (Figure 7B). 5meC DNA methylation is not directly involved in the imprinting process that causes silencing of the paternal chromosome set in coccid males. The paternal chromosome set is hypomethylated in both males (where the paternal chromosome set is silenced) and females (where both parental genomes are active) [53]. Instead, 5meC may protect the maternal genome from heterochromatinization in males.

Here, we show there are similarities between the imprinting system in *Sciara coprophila* and that observed in coccids and, in particular, that seen in mammals. Remarkably, we found that the sub-telomeric region adjacent to the X centromere, where the CE maps contain a unique enrichment of 5meC; we did not observe any similar 5meC signals elsewhere in the genome (Figure 3, Figure 4 and Figure 6, Supplementary Figure S2). The putative CE is also enriched for the repressive histone modification H3K9me3 and high-resolution ChIP-Seq profiles show that there is a precise overlap between 5meC and H3K9me3 in the 2.1Mb region that maps to the CE (Figure 4 and Figure 6). There is some overlap with the profile of the repressive histone modification H4K20me3 and clear depletion of the “active” histone modification H3K4me3 (Figure 4 and Figure 6). Based on these data and the observations that: (i) H4K20me3 is produced by H4K20 HMTases recruited by HP1 proteins bound to H3K9me3 [43,48] and, (ii), that H3K9me3, H3K20me3 and *Sciara* HP1 homologues co-localize to the CE on polytene chromosomes [5,20] we suggest that the H3K9me3/HP1/H4K20me3 pathway is involved in the mechanism of imprinting in *Sciara* as it does in coccids and mammals (c.f. Figure 7C with Figure 7A,B). Our data are consistent with the model (Figure 2), where the H3K9me3/HP1/H4K20me3 triad forms a heterochromatin-*like* complex that silences CE activity in the X chromosomes that avoid elimination. With the added parallel with mammals in that 5meC is part of the heterochromatin-*like* complex (c.f. Figure 7A with Figure 7C). Accordingly, the H3K9me3/HP1/H4K20me3 pathway may represent the most evolutionarily conserved part of the mechanism associated with imprinting in different species [5,19,45,46,48].

## 5. Conclusions

By defining a putative CE we can now undertake functional studies by introducing a CE-containing transgene into *Sciara* and testing whether integration of the CE into an ectopic chromosomal site leads to elimination of the chromosomes that harbour the transgene. This will provide an opportunity to study the mechanism by which the CE regulates elimination. Particularly, direct approaches should reveal if there is an ncRNA regulated by the CE, which is an important detail in the suggested mechanism of X chromosome elimination in *S. coprophila.* The stage is now set for the molecular dissection of chromosomal imprinting in *Sciara*, a century after the first studies on this fascinating system began.

## Data Availability

The data that support the findings of this study are available as BioProject PRJNA1271224 and BioProject PRJNA1273090 at https://www.ncbi.nlm.nih.gov/bioproject/ (accessed on 1 June 2025).

## Supplementary Materials

The following supporting information can be downloaded at: https://www.mdpi.com/article/10.3390/cells14161243/s1, Figure S1: 5meC immunostaining specificity control; Figure S2: DNA methylation staining is specifically localized to the presumptive CE region at the tip of the short arm of the X chromosome; Figure S3: Hi-C map of *S. coprophila* genome; Figure S4: Hi-C map of the sex chromosome in *S. coprophila*. References [55,56,57,58,59,60,61,62,63] are cited in Supplementary Materials.
