# Supplementary material for: DNA and Histone Modifications Identify a Putative Controlling Element (CE) on the X Chromosome of Sciara coprophila
Source: Cells. 2025 Aug 12;14(16):1243. doi: 10.3390/cells14161243 (PMC12385170; doi:10.3390/cells14161243)
Supplement: Supplementary file 1 [file cells-14-01243-s001.zip › cells-3714407-supplementary.pdf]

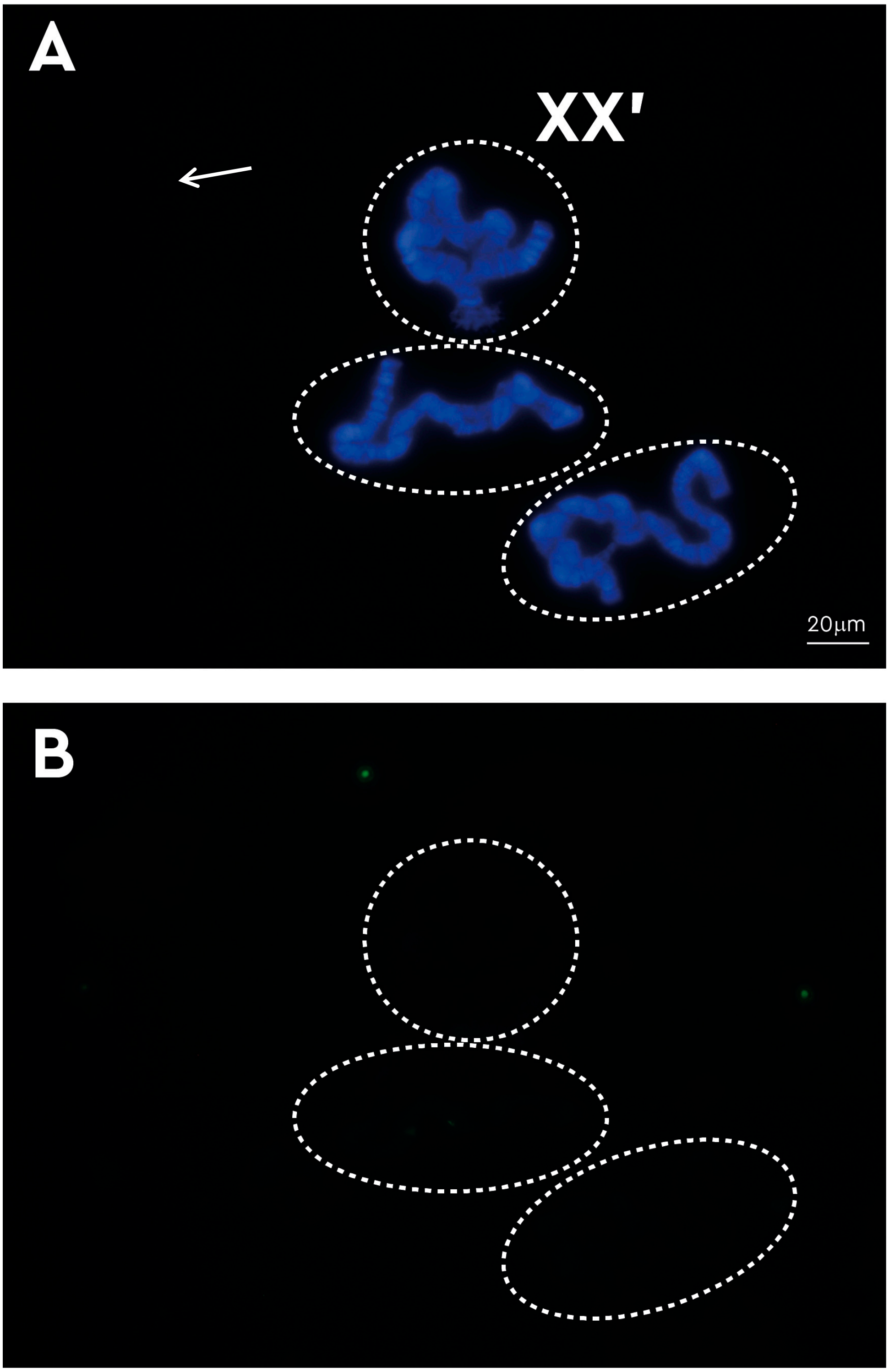

Supplementary Figure S1. 5meC immunostaining specificity control. A – DAPI staining. B – FITC fluorescence from the secondary antibodies conjugate. In absence of α-5meC antibodies no staining was observed. Photographs are presented as they were obtained without any adjustments. The XX' chromosomes and autosomes are outlined with dashed ovals.

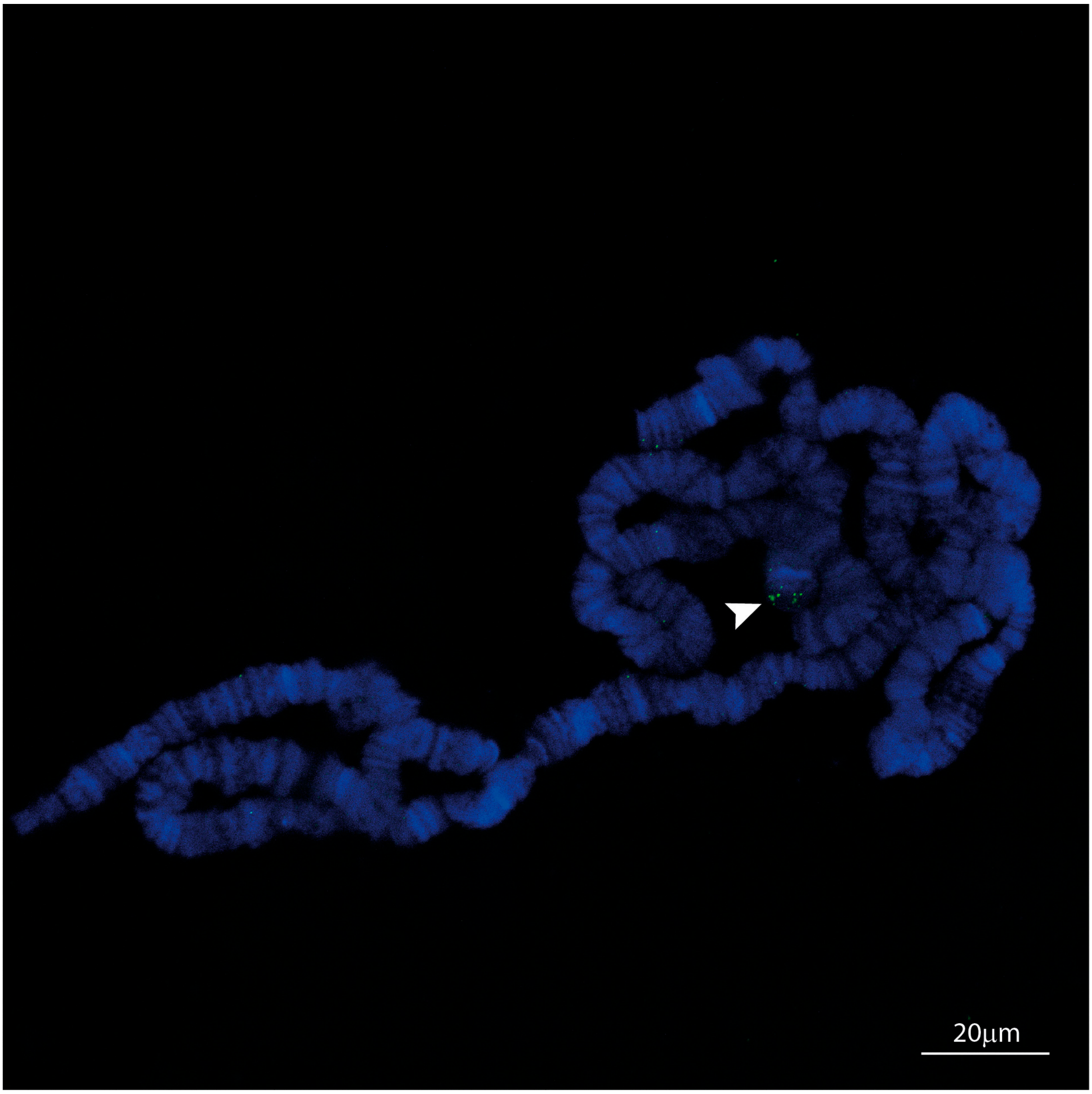

Supplementary Figure S2. DNA methylation staining is specifically localized to the presumptive CE region at the tip of the short arm of the X chromosome (indicated by the arrowhead). The image shows the entire squashed polytene nucleus.

Sciara coprophila *de novo* genome sequencing and assembly

Genome sequencing

Larval brains were dissected from Sciara coprophila XX and XX' females and used as a source of genomic DNA for sequencing. This DNA contained both X and X' chromosomes, so in the resulting data there were reads corresponding to both non-inverted X and inverted X' chromosomes. To perform genome assembly we generated 1.8 million Oxford Nanopore reads (approximately 54-fold genome coverage after filtering) and 120 million Illumina reads (2x75bp, approximately 32-fold genome coverage). The data are available at NCBI BioProject PRJNA1273090. Initial genome assembly based on Oxford Nanopore reads was performed using the Flye software [55]. This assembly was polished using Illumina reads using Pilon software [56]. The length of the resulting genome comprised about 333Mb.

Hi-C map generation.

Hi-C method for global analysis of chromatin conformation proved to be an extremely useful approach to improve genome assemblies enabling the correct ordering the contigs into chromosomes [57,58]. 3C library from adult XX' females was used for Illumina sequencing. The experiment was performed in 2 biological replicates. This procedure resulted in chromosome-level assembly of S. coprophila genome. Indeed, after Hi-C-enabled corrections the map clearly shows one metacentric chromosome (chromosome IV) and three acrocentric chromosomes (chromosomes X, II, and III) (Supplementary Figure S2). About 15% of smaller contigs remained outside the main four groups either due to their size or the insufficient quality.

X' chromosome inversion in the Hi-C maps

Apart from improving the integrity of the genome assembly Hi-C maps show the conformation of chromosomes within the nucleus as well as chromosomal rearrangements [59,60]. Our Hi-C from XX' adult females detected the distant contacts within the sex chromosome. Remarkably,

one of these contacts approximately corresponded to presumptive positions of the large inversion in X' chromosome as estimated from the cytological data. However it was also possible that this contact corresponds to a large chromosomal loop.

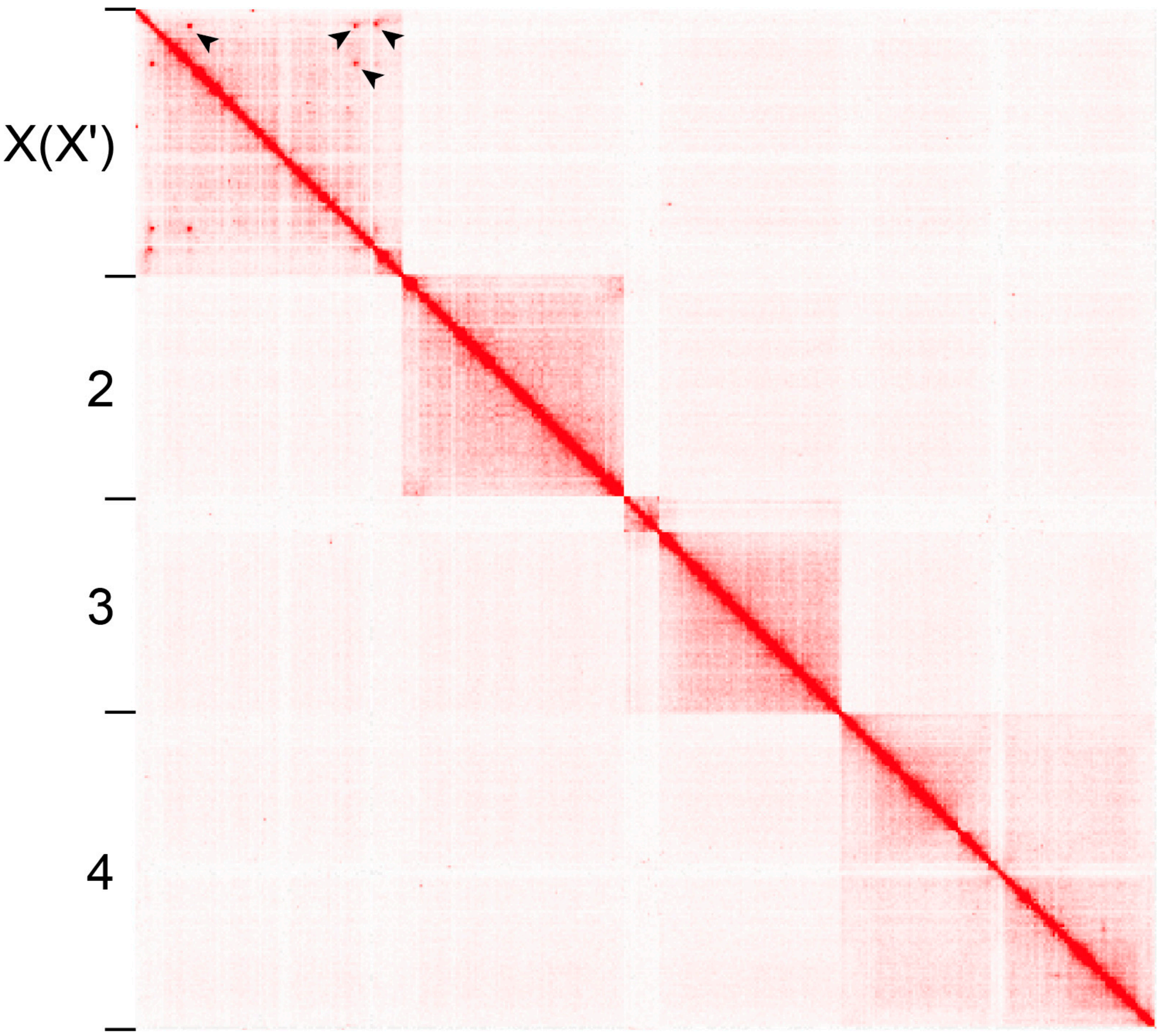

Supplementary Figure S3. Hi-C map of *S. coprophila* genome. Chromatin from XX' females was used to generate the 3C library. Chromosomes are indicated on the left. Arrowheads point out the distant contacts discovered in the sex chromosome

To distinguish between these two possibilities we generated Hi-C map from adult males (XO). This material did not contain X' chromosome. Comparison of two Hi-C maps showed that the contact observed in XX' females completely disappears in XO males. This clearly indicates that this contact corresponds to the inversion in the X' chromosome (Supplementary Figure S3). This picture correlates very precisely with recently published Hi-C maps of *S. coprophila* X and X' chromosomes [15].

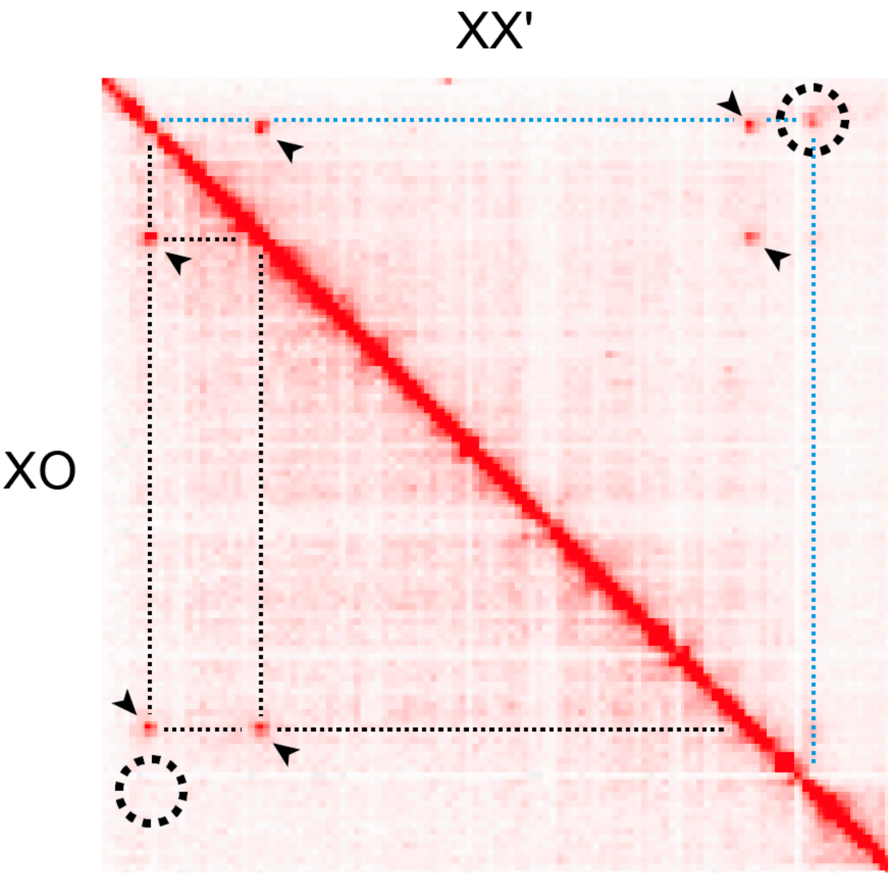

Supplementary Figure S4. Hi-C map of the sex chromosome in *S. coprophila*. Above the diagonal – Hi-C contact map in XX' adult females; below the diagonal – in adult XO males. Chromatin loops are shown with the black dotted lines, the contacts formed by the bases of these loops are shown with the arrowheads. The inversion in the X' chromosome is shown with the blue dotted line. The concentration of the hybrid reads at the inversion breakpoints is circled on the female Hi-C map; no hybrid reads is observed at the same location in XO males lacking the inversion.

Supplementary Methods

Genome sequencing

Genomic DNA was isolated from larval brains as previously described [61]. Libraries for Illumina sequencing on Oxford Nanopore and Illumina sequencers were prepared as recommended by the manufacturers. Initial genomic assembly was performed from the Nanopore long reads using Flye software [55] with the default settings. On the next step Pilon software was used to correct the sequencing errors and improve the assembly [56].

In situ Hi-C

For each Hi-C experiment, ten adult gnats were homogenized using a pre-cooled Dounce homogenizer in PBS with 2% formaldehyde for 2 minutes. Suspension was filtered through a 40 µm cell strainer. Cells were fixed for 10 minutes at RT, then glycine was added to 0.125 M final concentration and incubated for 10 minutes at RT. Suspension of fixed cell was centrifuged for 10 minutes at 2,500 × g, and supernatant was discarded. Pellet was washed with PBS, centrifuged again, frozen in liquid nitrogen and stored at −80 °C.

On the day of the Hi-C experiment, the cell pellet was thawed at RT, resuspended in 1 mL of insect cell lysis buffer (150 mM Tris-HCl pH 8.0, 140 mM NaCl, 0.5% IGEPAL CA-630, 1% Triton X-100, 1x protease inhibitor cocktail) and incubated for 45 minutes with slow rotation. The rest of the protocol was proceeded according to the method described previously [62] with minor modifications: nuclei were treated with 0.3% SDS in NEB 3.1 buffer for 1 hour at 37 °C, DpnII (NEB) was used for chromatin digestion and centrifugation force was increased to 5000 x g for pelleting nuclei, digested and re-ligated chromatin. Libraries were prepared using KAPA HyperPlus kit (Roche) and sequenced in paired-end 150 bp mode on Illumina platform. To visualize the resulting Hi-C maps the Juicer software was used [63]. The data are available as BioProject PRJNA1271224.
